# Development and evaluation of a new, specially tailored CHO media platform

**DOI:** 10.1186/1753-6561-7-S6-P35

**Published:** 2013-12-04

**Authors:** Tim F Beckmann, Christoph Heinrich, Heino Büntemeyer, Stefan Northoff

**Affiliations:** 1 TeutoCell AG, Bielefeld, 33613, Germany

## Background

Today's biopharmaceutical industry is under increasing pressure considering cost efficient development. Short timeframes rule the progress starting from the generation of producer cell lines to the establishment of a final production process. Hence, the timescale for optimization of cell culture media is small, but on the other hand it contains high potential for global process improvement. In this scope, our specially tailored media development platform, which allows a fast and reliable introduction of high-performance basis media and feeds, establishes new perspectives for an efficient process development.

## Materials and methods

For the design and development of TeutoCell's new media platform various cell lines and expression systems were comprehensively analyzed and incorporated. The results gained from cultivations and extensive analysis of culture supernatant and e.g. product glycosylation were integrated in a cyclic development strategy, utilizing theoretical and empirical formulation optimizations. Special applications like single clone selection were integrated into our platform as well.

The cell lines used for the development of our media platform include CHO-DG44, CHO-GS and CHO-K1 clones. Cultivations were carried out in shaking flasks as well as closed-loop controlled 0.5 - 2.0 L bioreactor systems in batch und fed-batch mode using standard conditions. An industrially relevant, protein-free and chemically defined medium was used as a reference.

Media development for single clone selection by limited dilution was performed with different CHO suspension cells in microtiter plates (from 96- to 6-wells) up to shaking flaks. Analysis of single clone colonies was done with a Cellscreen System.

Samples of a model antibody produced in commercially available CHO reference medium and TeutoCell's platform medium using two different producer clones were desalted, denaturated and treated with PNGaseF. Glycans were concentrated via solid-phase extraction and analyzed by MALDI-TOF mass spectrometry. Signal-to-noise ratios of specific masses were used for calculations of relative amounts.

## Results

**Figure 1:**
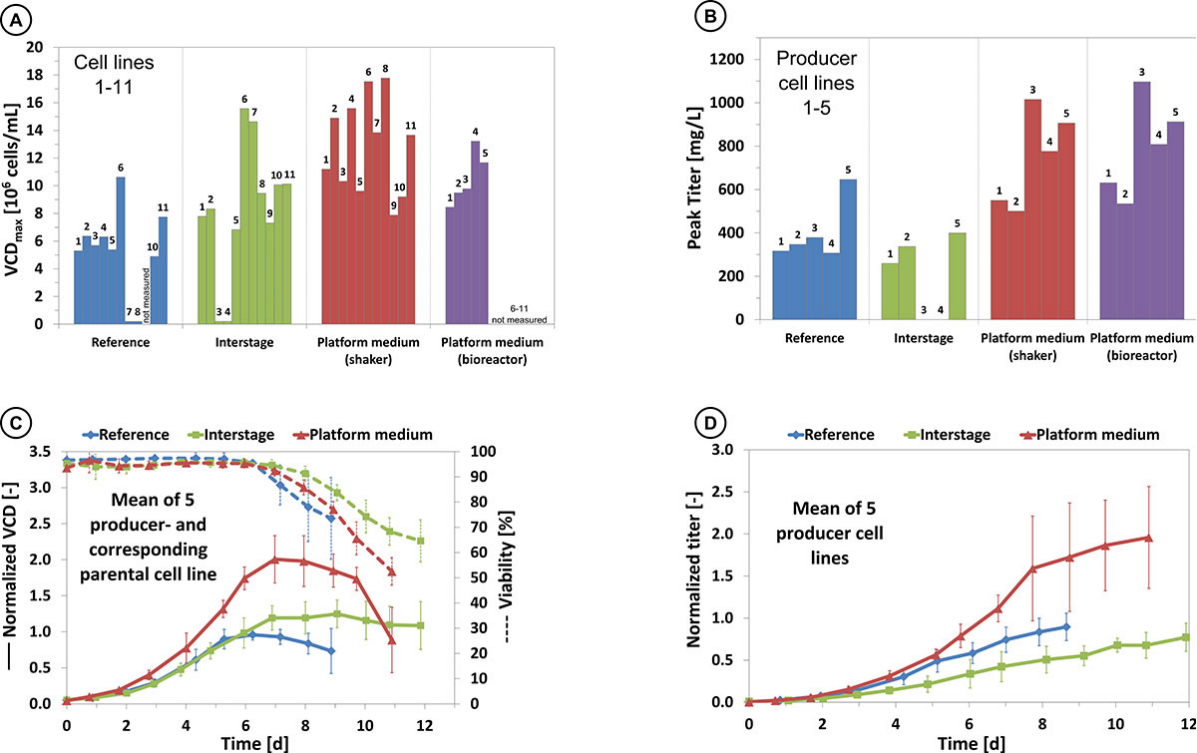
**Comparison of growth performance **(A) **and product titer **(B) **using the reference and platform medium**. To illustrate the improvement in relation to the reference medium, the mean of 5 producer- and the corresponding parental cell line were normalized to the results obtained in the reference medium (**C **and **D**). The error bars show the deviation between the different cell lines. The development progress of the platform medium is represented by one major interstage.

The performance of the platform medium was evaluated using a set of eleven different CHO cell lines in comparison to an industrially relevant, protein-free and chemically defined medium. For all tested cell lines, the maximum viable cell density (vcd) as well as the integrated viable cell density (ivcd) and the product titer were higher compared to the reference. In numbers, the improvement in vcd ranged between a factor of 1.7 and 2.5, in ivcd between a factor of 1.2 and 4.2 and in product titer between a factor of 1.4 and 2.7 in batch cultures. By this improvement viable cell densities of up to 17.53·10^6 ^cells/mL and product titer of 1015 mg/L were reached. An overview of these results is illustrated in Figure 1.

Furthermore, the potential influence of the utilized medium on product glycosylation was examined. For this, antibody harvest from two different clones cultivated in reference and platform medium was analyzed. The results of relative quantification of glycan structures by mass spectrometry showed highly comparable profiles for the reference and platform medium. An overview of the glycoanalysis is given in Table 1.

**Table 1:** Comparison of the glycosylation pattern of a model antibody produced in two different cell lines using the reference medium and the platform medium.

Structure	Relative Amount of Glycan Structure [%]					
	
	High Producer 1			High Producer 2		
	
	Reference Shaker	Platform Shaker	Platform Bioreactor	Reference Shaker	Platform Shaker	Platform Bioreactor
Man3	-	-	-	4 ± 2	1 ± 2	-
Man5	7 ± 2	3 ± 2	5 ± 2	12 ± 3	9 ± 2	4 ± 1
G0F-GlcNAc	2 ± 2	2 ± 1	4 ± 2	10 ± 4	5 ± 0	2 ± 2
G0F	46 ± 2	50 ± 5	47 ± 5	47 ± 5	47 ± 2	47 ± 5
G1	6 ± 3	6 ± 2	8 ± 2	3 ± 1	9 ± 1	5 ± 1
G1F	30 ± 3	32 ± 5	29 ± 7	19 ± 2	23 ± 3	35 ± 2
G2F	9 ± 2	7 ± 2	7 ± 2	5 ± 1	6 ± 1	7 ± 2

The relative amounts of detected structures are given in % with the standard deviation of four independent analyses.

As an additional application, the platform medium was successfully utilized as a basis for a chemically defined cloning medium in limited dilution experiments. For different cell lines single cell growth was achieved and cells were effectively expanded from 96-well plate format up to shaking flask cultures.

## Conclusions

Within this work a chemically defined and animal-component free media platform was successfully implemented, which supports high performance growth and productivity without supplementation of proteins or growth hormones. In addition, its streamlined formulation of less than 50 components increases the design space for the efficient development of custom formulations. The suitability as a platform medium was verified by the successful cultivation of a wide range of cell lines including CHO-DG44, CHO-GS and CHO-K1 clones and the feature of easy adaption from serum containing and commercially available formulations. For all tested cell lines stable high performance cultivations with high product yields were achieved, with consistent glycosylation profiles. As a further field of application, the platform medium provides the basis for single cell growth following limited dilution.

